# Large B-cell Lymphoma-Associated Membranous Nephropathy With Positive PLA2R on Kidney Biopsy

**DOI:** 10.7759/cureus.48902

**Published:** 2023-11-16

**Authors:** Mina Al-Khazraji, Inam A Al-Mufti, Yamama Al-Khazraji

**Affiliations:** 1 Internal Medicine, Royal Bolton Hospital, Bolton, GBR; 2 Chemistry, Al-Qalam University College, Kirkuk, IRQ; 3 Nephrology, Clinical Research Consultants, Kansas, USA

**Keywords:** anti-phospholipase a2 receptors, large b-cell lymphoma, primary membranous nephropathy

## Abstract

Large B-cell lymphoma associated with membranous nephropathy (MN) is a rare and complex medical condition that involves the simultaneous presence of two distinct diseases: a malignant lymphoma and a kidney disorder called membranous nephropathy. In this case, there is an additional element of interest, which is the presence of positive Phospholipase A2 Receptor (PLA2R) in the kidney.

The case involves a 53-year-old Caucasian male with a three-week history of lower leg edema and a past medical history of recurrent upper respiratory infections. The upper respiratory infections were characterized by symptoms of fever, sore throat, and headache, and they required multiple rounds of antibiotics for treatment, including Augmentin and Keflex. A diagnosis of nephrotic syndrome was made based on proteinuria of fourteen grams with no RBC cast on urinalysis. Kidney biopsy stained positive for antiposophlipase A2 receptor on a frozen section. Thrombospondin type 1 domain-containing 7A (THSD7A) was not detected.

Given his biopsy and absence of clinical symptoms, he was treated as a case of primary membranous nephropathy with angiotensin-converting enzyme (ACE) inhibitors, steroids, and immunosuppressive. Three months following the treatment, his condition deteriorated, and after a thorough investigation, he appeared to have large B-cell lymphoma as a secondary cause of membranous glomerulopathy (MGN).

When large B-cell lymphoma is associated with membranous nephropathy, it presents a unique clinical challenge. The interaction between these two conditions is not fully understood, but it is believed that the lymphoma may provoke an immune response that leads to the development of membranous nephropathy. Moreover, the presence of positive PLA2R in the kidney indicates a specific mechanism at play in this complex disease scenario. Treatment for this condition typically involves addressing both the lymphoma and the kidney disorder. This may include chemotherapy or other treatments to target the lymphoma and immunosuppressive therapy to manage the autoimmune response causing membranous nephropathy. Close monitoring and coordination between oncologists and nephrologists are essential for the best possible outcome in managing this rare and challenging condition.

## Introduction

Membranous nephropathy (MN), also known as membranous glomerulopathy, is the most common cause of nephrotic syndrome in adults and occurs as an idiopathic (primary) or secondary disease. It is characterized by massive proteinuria (>3.5 g/day) and clinically presents with peripheral edema, puffy eyes, weight gain, foamy urine, hypertension, and manifestations of thromboembolic phenomena [1].

A 53-year-old male with nephrotic syndrome underwent renal biopsy which showed features of membranous glomerulopathy (MGN) on light and electron microscopy. Immunohistochemical staining for phospholipase A2 receptor was performed on the frozen tissue and showed diffuse positive staining in the glomeruli which suggested primary membranous glomerulopathy. Immunofluorescence showed that the sections are stained for IgG, IgM, IgA, C3, C1q, albumin, fibrinogen, and kappa and lambda light chains. The renal parenchyma submitted consists of 10% of the cortex.

He was treated with corticosteroid and immunosuppressive after conservative therapy had failed. After his third month of treatment he developed hypoxic respiratory failure and later was diagnosed with stage IV large B-cell lymphoma.

## Case presentation

**Figure 1:**
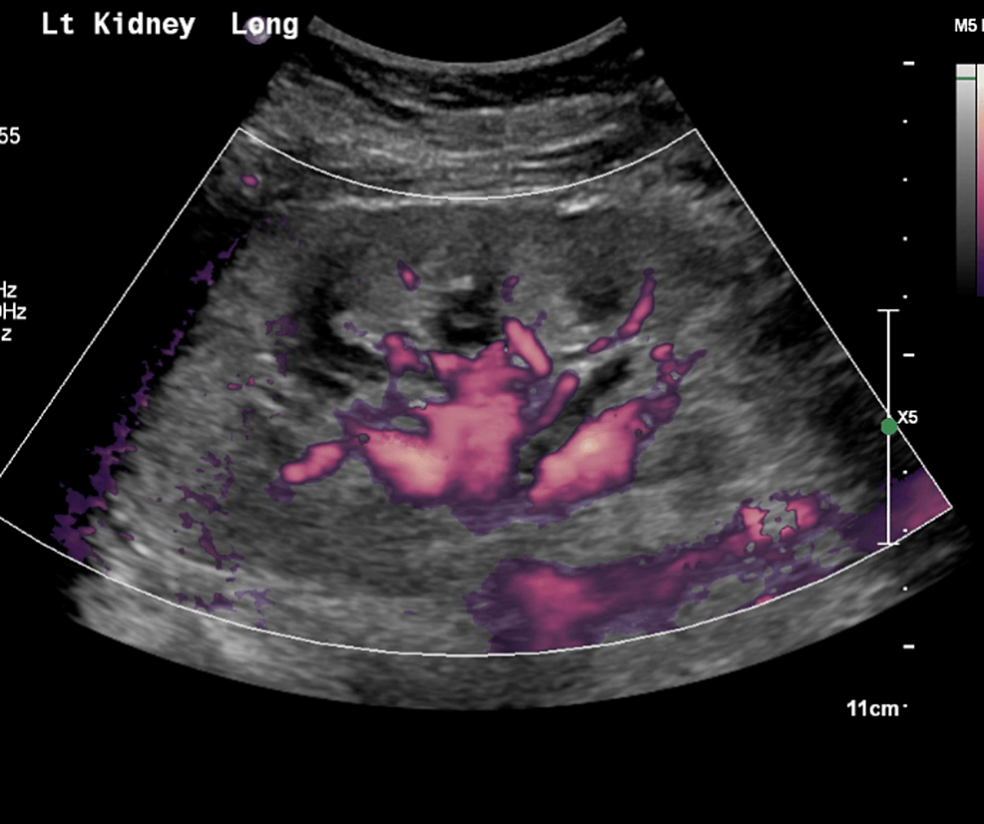
Doppler ultrasound of left kidney: Increased renal echogenicity, consistent with medical renal disease.

**Figure 2:**
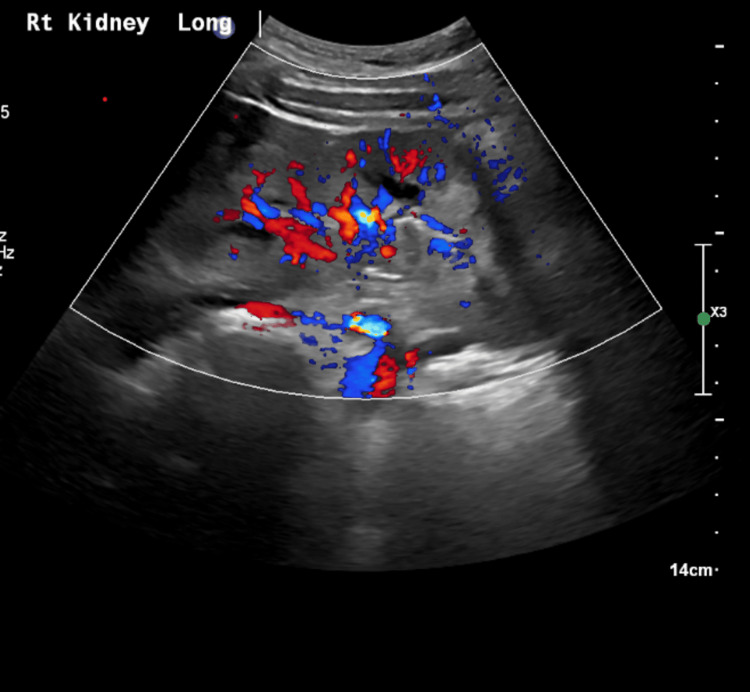
Doppler ultrasound of right kidney: Increased renal echogenicity, consistent with medical renal disease.

A 53-year-old Caucasian male with a history of upper respiratory tract infection for two months treated with several rounds of antibiotics, including Augmentin and Keflex; presented with lower leg edema, and anasarca, his serum creatinine was 1.3 mg/dl, eGFR 70 ml/min/1.73, protein/creatinine ratio 7386mg/gm., total serum protein 5gm/dl, Albumin 2.4 gm/dl, complement levels were normal, doppler ultrasound of the right and left kidney showed increased renal echogenicity, consistent with medical renal disease (Figures 1, 2). The chest x-ray was unremarkable. He was diagnosed with nephrotic syndrome. A renal biopsy was performed, the microscopic examination was consistent with membranous nephropathy, and ant-PLA2R antibodies tested positive, suggesting active primary membranous nephropathy. Thrombospondin type 1 domain-containing 7A (THSD7A) was not detected on the biopsy. He was treated as primary MGN based on positive serum PLA2R and the absence of clinical symptoms. The patient received Losartan 25 mg. Three days later, he experienced a complex partial seizure. His EEG showed generalized periodic epileptiform discharge, indicating a tendency toward seizure and triphasic waves related to his metabolic derangement (Na of 122 mg/dl, Ca of 7.8 mg /dl, and magnesium of 1.8 mg/dl), likely from the nephrotic syndrome. He was treated with Depakote and correction of the electrolyte. His brain MRI without contrast was normal, cardiac Echo was normal with EF of 65%, and colonoscopy was normal. The patient started immunosuppressive therapy consisting of cyclophosphamide and prednisolone. On the 3rd month of treatment, he developed hypoxic respiratory failure manifested as shortness of breath, dizziness, and confusion.

MRI of the brain with contrast showed two small foci of restricted diffusion in the right cerebellum and occipital lobe. MRI of the abdomen with contrast showed bilateral adrenal gland lesions demonstrating restricted diffusion, upper abdominal lymphadenopathy, bilateral pleural effusion, and splenomegaly. A lymph node biopsy of multiple regions was performed and revealed a large B-cell lymphoma. The patient was diagnosed with stage IVB diffuse large B-cell lymphoma with involvement of adrenal glands and bone marrow with the complicated course of encephalopathy, membranous nephropathy, and sepsis International Prognostic Index (IPI) score was 4. FISH has a gain of chromosome 11,14q32.3 (IGH) and 18q21(BCL2) in 3.2% of nuclei and a gain of 8q24(MYC) in 2% of nuclei. He was admitted to receiving ten cycles of DA- EPOCHR(Etppoiside+Prednisolone+Vincristine+Cyclophosphamide+Doxorbucin+Rituxmab. Cycle one was given daR-REPOCH with intrathecal Methotrexate for CNS prophylaxis. The course was complicated with acute kidney injury, tubular necrosis, and membranous nephropathy, requiring dialysis. He had significant lower leg edema, and occupational therapy helped with that. He had anemia with thrombocytopenia from chemotherapy. Cycles two and three went well. Cycle five was complicated with neutropenic fever due to viral infection and oral thrush. After six cycles of chemotherapy, his PET CT was consistent with a metabolic complete response. However, it showed a hyperdense left external iliac chain lymph node, which has been unchanged in size since the start of his treatment. He had bilateral lower extremity swelling due to his membranous nephropathy. The patient's neuropathy has improved, and he was prescribed Neuropogen for his agranulocytosis. His protein/creatinine ratio is 630 mg/gm.

## Discussion

Our patient's biopsy had features of MGN with 20% cortex and 80% medulla involvement. Up to five glomeruli are present, one of which is globally sclerotic. There is diffuse mild interstitial edema associated with a patchy interstitial mononuclear infiltrate within the cortex and corticomedullary junction. Diffuse positive staining in the glomeruli for phospholipase A2 receptor (PLA2R) on the frozen section in case of nephrotic syndrome with 14 g of proteinuria on 24‐hour urine collection. He had no signs and symptoms suggestive of secondary MGN. His antinuclear AB (ANA), antineutrophilic cytoplasmic antibody (ANCA), Hepatitis B, and C complements were normal.

In patients with nephrotic syndrome, the anti-PLA2R1 antibodies are linked with the diagnosis of primary membranous nephropathy according to a recent meta-analysis (all study sensitivity 78% (95% CI: 66% to 87%) and specificity 99% (95% CI: 96% to 100%)) [2]. The detection of anti-pla2r1 antibodies is counted for approximately 70-80% of patients with idiopathic membranous nephropathy in Europe [3], USA [4], Asia [5]. while in Japan these antibodies are in 53% of patients with idiopathic MN, which is lower than in other Asian countries [6,7].

The prognosis of patients with membranous nephropathy varies, however about 30 to 40% end up with end-stage kidney disease within five to 15 years of diagnosis [8]. Primary membranous nephropathy is responsible for 75% of the cases of membranous nephropathy and 25% due to secondary causes such as autoimmune (systemic lupus arthritis), infection (hepatitis), drugs (gold salt and NSAID) and malignancy (lymphoma) [9]. The main pathogenesis of the disease is complement activation which results from the deposits of an immune complex on the outer surface of the glomeruli basement membrane which has circulating antibodies and antigens [10]. The immune complex forms from the binding of these circulating antibodies to one of the two naturally- expressed podocyte antigens: the M-type receptor for secretory phospholipase A2 and thromboplastin type 1 domain-containing 7A (THSD7A) [11]. Autoantibodies to PLA2R1 at presentation are helpful for diagnosis and monitoring of the disease. The present case was diagnosed as Primary MN at the initial onset as the renal biopsy stained positive for phospholipase A2 receptor (PLA2R). However, the patient's condition did not respond to a trial of conservative therapy and corticosteroid and was diagnosed later with diffuse large B-cell lymphoma stage IV as a secondary cause of membranous nephropathy. Membranous nephropathy associated with malignant neoplasm is considered a paraneoplastic syndrome (PNS) or paraneoplastic glomerulopathy [12].

## Conclusions

This case report represents nephrotic syndrome due to secondary membranous nephropathy with kidney biopsy stained for phospholipase A2 receptor on a frozen section. Patient was treated initially as a case of primary MGN based on positive PLA2R on kidney biopsy and absence of clinical symptoms suggestive of secondary MGN. He showed no clinical response to ACE inhibitors and steroid. After three months his condition deteriorated and he was diagnosed with large B-cell lymphoma.
